# Vertically Well-Aligned ZnO Nanoscintillator Arrays with Improved Photoluminescence and Scintillation Properties

**DOI:** 10.3390/ma16206717

**Published:** 2023-10-17

**Authors:** Murat Kurudirek, Sinem V. Kurudirek, Nolan E. Hertel, Anna Erickson, Paul J. Sellin, Sharmistha Mukhopadhyay, Aykut Astam, Christopher J. Summers

**Affiliations:** 1 Nuclear and Radiological Engineering, Georgia Institute of Technology, Atlanta, GA 30332, USA; sinem.kurudirek@me.gatech.edu (S.V.K.); nolan.hertel@me.gatech.edu (N.E.H.); anna.erickson@me.gatech.edu (A.E.); sharmistha.mukhopadhyay@me.gatech.edu (S.M.); 2 Department of Physics, University of Surrey, Guildford GU2 7XH, UK; p.sellin@surrey.ac.uk; 3 Department of Electricity and Energy, Technical Sciences Vocational College, Ataturk University, Erzurum 25240, Turkey; 4 Department of Physics, Faculty of Arts and Science, Erzincan Binali Yıldırım University, Erzincan 24100, Turkey; aastam@erzincan.edu.tr; 5 School of Materials Science and Engineering, Georgia Institute of Technology, Atlanta, GA 30332, USA; chris.summers@mse.gatech.edu

**Keywords:** nanoarrays, scintillators, hydrothermal method, ZnO

## Abstract

ZnO nanoarrays were grown via a low-temperature hydrothermal method. Solutions, each with different additive combinations, were prepared and evaluated. The effects of the additives involved in the growth procedure, i.e., ammonium hydroxide and sodium citrate, were studied in terms of the morphological, optical and scintillation properties of the ZnO nanostructures. Measurement of the nanorod (NR) length, corresponding photoluminescence (PL) and scintillation spectra and their dependence on the additives present in the solution are discussed. ZnO NRs grown on a silica substrate, whose UV transmission was found to be better than glass, showed high-quality structural and optical properties. It was found that the addition of sodium citrate significantly reduced defects and correspondingly increased the intrinsic near-band-edge (NBE) UV emission intensity at ~380 nm. To obtain high-quality nanostructures, samples were annealed in a 10% H_2_ + 90% N_2_ atmosphere. The anneal in the forming gas atmosphere enhanced the emission of the UV peak by reducing defects in the nanostructure. NRs are highly tapered towards the end of the structure. The tapering process was monitored using time growth studies, and its effect on PL and reflectance spectra are discussed. A good alpha particle response was obtained for the grown ZnO NRs, confirming its potential to be used as an alpha particle scintillator. After optimizing the reaction parameters, it was concluded that when ammonium hydroxide and sodium citrate were used, vertically well-aligned and long ZnO nanoarrays with highly improved optical and scintillation properties were obtained.

## 1. Introduction

The synthesis of nanomaterials with different morphologies has always been a subject of research in the field of material science and its applications, viz., photonics, electronics and optoelectronics. Its noncentral symmetric structure and large electrochemical coupling leads to strong piezoelectric and pyroelectric properties, and its polar surfaces promote anisotropic growth of many interesting nanostructures [1]. Due to its unique properties, it is being used and investigated for various applications such as piezoelectric transducers, optical waveguides, surface acoustic wave devices, transparent conductive oxides, chemical and gas sensors, solar cells, ultraviolet lasers and light emitting diodes and photodetectors [2,3,4,5,6,7,8,9,10,11,12,13,14,15,16,17]. Its wide and direct bandgap and large exciton binding energy make ZnO a promising candidate for an efficient emitter. In addition, ZnO possesses excellent properties related to scintillation applications, such as relatively high stopping power (with density (5.7 g.cm^−3^) and effective atomic number (28)), fast fluorescence lifetime of excitonic decay (ps to ns), high NBE UV emission (~380 nm) and radiation tolerant thermal and chemical stability [18,19,20,21,22,23]. Furthermore, its high melting point and fast scintillation properties make it particularly well-suited for use as a fast alpha particle scintillator. This is especially important when simultaneously detecting alpha particles and neutrons in D-T sources [24,25].

While the physical and chemical properties of bulk materials are well-established and predictable, they undergo variations and become less predictable at nanoscale levels [26,27,28,29]. The controlled synthesis and the optimization of the growth procedures for nanoscaled materials become of vital importance. Many efforts have been made to grow 1D ZnO nanomaterials like quantum dots, wires, rods and tubes [30].

Various methods have been explored for ZnO nanomaterial synthesis, for example, metal–organic chemical vapor deposition (MOCVD), pulsed laser deposition, chemical vapor transport, electrodeposition, radio-frequency magnetron sputtering, molecular beam epitaxy and hydrothermal synthesis [31,32,33,34,35,36,37,38,39,40,41,42]. These methods have some limitations on the material, substrate and operational temperature. They either require single-crystal or conductive substrates, and the growth temperature may reach up to 950 °C. On the other hand, hydrothermal growth has some advantages as a low temperature and conventional equipment are used for material growth, and it is of a relatively low cost when compared to the above methods.

In the solution-based growth of ZnO NRs, generally a zinc source, such as zinc nitrate or zinc acetate, and hexamethylenetetramine are used as raw materials. However, this route ends up with defect-related visible emissions in addition to the near-band-edge UV emission. To gain greater control over the growth and resulting NR morphology, and to suppress the visible emission in the structure, ammonium hydroxide and citrate were used in the present work as additives to this basic solution. Different reaction parameters such as precursor concentrations, synthesis time and temperature, substrate, seed layer and zinc source, etc., strongly effect the size and structure of the ZnO NRs. In this study, we demonstrate for the first time how the reaction parameters were optimized for solutions with additives (as mentioned above), resulting in an enhancement in crystalline quality, as well as in optical and scintillation properties of ultralong and ultradense ZnO nanoarrays.

This study is aimed at the growth of high-quality ultralong ZnO NRs using a low-cost hydrothermal method. Citrate has been included in the growth process as an additive to suppress the defect-related emission and to control the morphological, optical and scintillation properties of these materials. To improve the quality of the NRs, samples were annealed in a forming gas. Tapered NRs were obtained and the effect of tapering on the PL and reflectance is discussed.

## 2. Materials and Methods

**Figure 1:**
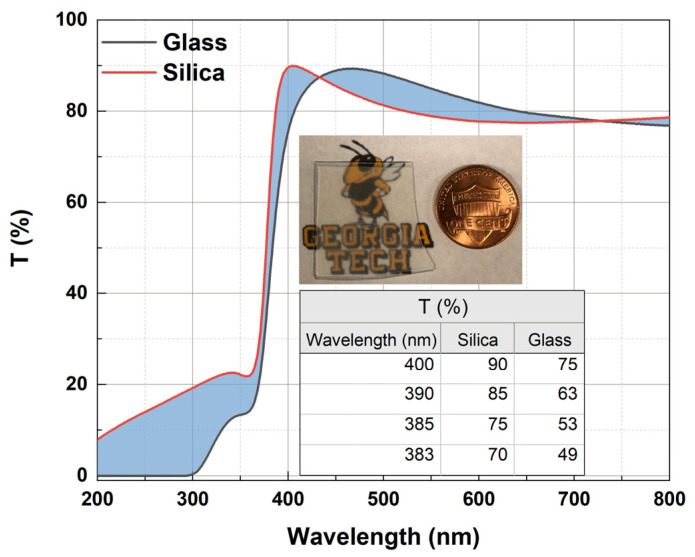
UV–Vis transmission spectra of the silica and glass substrates with a 100 nm thick ZnO seed layer. Shown (top inset) is a typical ZnO NR sample grown on a silica substrate. Inset table indicates the transmission (%) over the same UV region as the NBE UV emission of ZnO.

ZnO NRs were grown on 0.5 mm thick silica substrates (MSE Supplies, Tucson, AZ, USA) prepared with a sputtered ZnO seed layer. An RF sputtering technique with Ar ion bombardment of the ZnO target was used to obtain the 100 nm thick ZnO seed layer. Samples were annealed in a forming gas atmosphere containing 10% H_2_ for 1 h at 350 °C. Silica glass substrates were chosen due to their higher transmission values when compared to glass, especially in the UV region (Figure 1). The optimum reaction time and temperature were 25 h and 95 °C, respectively. Time growth studies were performed in the range of 3 h to 27 h wherever possible. The reactants were 0.05 mol/L Zn(NO_3_)_2_.6H_2_O (Sigma Aldrich, St. Louis, MO, USA, 98%) and 0.025 mol/L C_6_H_12_N_4_ (Sigma Aldrich, St. Louis, MO, USA, ≥99%). Na_3_C_6_H_5_O_7_ (Sigma Aldrich, St. Louis, MO, USA, ≥99%) and ammonium hydroxide (NH_4_OH, Sigma Aldrich St. Louis, MO, USA) were used as additives in the solution. Various molar concentrations of the trisodium citrate (0.6 mM, 0.9 mM, 1.2 mM, 1.5 mM and 1.8 mM) and the ammonium hydroxide (0.2 M, 0.5 M, 0.8 M, 1.1 M and 1.4 M) were studied. All chemicals were of analytical grade. A series of solutions were prepared using deionized water. For the series of solutions, the molar ratio (MR) of zinc nitrate to HMTA was maintained at 2:1. The synthesized materials were rinsed with DI water to make sure there was no residual salt or amino complex remaining on the surfaces and were kept at room temperature.

The structural properties of the materials were characterized using SEM (HITACHI SU8230, Tokyo, Japan) and XRD (Panalytical XPert PRO), and the optical properties were studied using a He-Cd 325 nm UV laser (KIMMON KOHA, Tokyo, Japan) and a PL spectrometer (Renishaw, Wotton-under-Edge, UK). The post-growth annealing was conducted in an annealing furnace (Thermolyne 21100 Tube Furnace, ThermoFisher Scientific, Waltham, MA, USA). The UV–visible spectra were collected using a Cary 5000 UV/Vis/NIR spectrophotometer (Agilent, Santa Clara, CA, USA). Scintillation measurements were performed using an Am-241 alpha source (emitting ~5.5 MeV alpha particles) and a PMT readout detector.

## 3. Results and Discussion

By using citrate and ammonium hydroxide as additives, we were able to grow long and well-aligned hexagonal NRs in one growth cycle, whereas previously, at least three growth cycles were needed as reported in the literature. Although the early mentioned nanomaterials showed a broad visible (defect) emission band, the UV peak increased and the visible emission band decreased when annealed in forming gas.

In many studies, sodium citrate has been used as an additive to obtain nanoparticles. However, studies in which well-aligned NRs were obtained using sodium citrate as an additive are scarce. Several studies by the authors of this paper were conducted earlier using citrate as an additive [25,43,44]. As a sequel to the previous works, the materials crystal quality and optical and scintillation properties have significantly improved in the present work by optimizing reaction parameters and using a different substrate. Using citrate and ammonium hydroxide as additives, we were able to grow well-aligned NRs (20 microns long) exhibiting a strong UV peak and negligible visible emission band. Also, after annealing these nanomaterials we noticed a huge change in the PL emission spectra such that the intensity of the UV peak increased by a factor of ~50, thus, becoming very strong while the visible emission band remained negligible. Following this procedure, we were able to obtain high-quality tapered NRs in one synthesis cycle.

### 3.1. Growth Process

Below are the fundamental chemical reactions taking place during the growth:

(1) $$\mathrm{Zn}{(NO_{3})}_{2}\cdot 6H_{2}O_{\left(s\right)}\to {Zn}_{\left(aqs\right)}^{2+}+2NO_{\left(aqs\right)}^{-}$$

(2) $$C_{6}H_{12}N_{4}+{6H}_{2}O\to 6\mathrm{HCHO}+4NH_{3}$$

(3) $$NH_{3}+H_{2}O\to {{\mathrm{NH}}_{4}}^{+}+OH^{-}$$

(4) $${\mathrm{Zn}}^{2+}+4{\left(\mathrm{OH}\right)}^{-}\to \;\mathrm{Zn}{\left({\mathrm{OH}}_{4}\right)}^{2-}$$

(5) $$\mathrm{Zn}{{\left(\mathrm{OH}\right)}_{4}}^{2-}\to \mathrm{ZnO}+H_{2}O+2{\left(\mathrm{OH}\right)}^{-}$$

(6) $${\mathrm{Na}}_{3}C_{6}H_{5}O_{7}\to {3\mathrm{Na}}^{+}+C_{6}H_{5}{O_{7}}^{3-}$$

(7) $${\mathrm{Zn}}^{2+}+C_{6}H_{5}{O_{7}}^{3-}\to {\mathrm{Zn}}^{2+}-\mathrm{citrate}$$

(8) $${\mathrm{Zn}}^{2+}-\mathrm{citrate}+NH_{3}+H_{2}O\to {\mathrm{Zn}}^{2+}+{{\mathrm{NH}}_{4}}^{+}+OH^{-}+C_{6}H_{5}{O_{7}}^{3-}$$

The primary source of zinc ions in the solution is the zinc nitrate (Equation (1)). HMTA is responsible for the continuous growth of ZnO by supplying OH^−^ in the solution in a two-step process in which the OH^−^ ions are released after the NH_3_ molecules hydrolyze following the dissolution of HMTA (Equations (2) and (3)). Thereafter, $\mathrm{Zn}{\left({\mathrm{OH}}_{4}\right)}^{2-}$ (aq) hydroxyl groups are formed by the reaction of Zn^2+^ with OH^−^ (Equation (4)) [45]. Following the dehydration of the hydroxyl species, ZnO nucleation is initiated and deposits on the surface on the substrate (Equation (5)). The vertically well-aligned ZnO NRs clearly confirm the growth along the c-axis. This is because ZnO has a positive facet (001) having the highest surface energy compared to the other facets, thus, promoting the fastest growth rate along this axis [46]. Equations (6)–(8) denote the reactions when additives like ammonium hydroxide and sodium citrate are used in the solution. After the citrate ions are formed in the solution by the dissolution of sodium citrate, Zn^2+^-citrate groups form (Equations (6) and (7)). Thus, together with the addition of ammonium hydroxide, citrate ions exchange with OH^−^ ions and contribute to ZnO growth by forming $\mathrm{Zn}{\left({\mathrm{OH}}_{4}\right)}^{2-}$ (aq) hydroxyl groups. Additionally, it has to be noted that the growth of ZnO nanomaterials depends strongly on concentration, growth temperature and growth time.

To optimize the reaction parameters, a series of solutions with different additive concentrations for growths at different growth times were prepared. First solutions with different concentrations of ammonium hydroxide (0.2 M, 0.5 M, 0.8 M, 1.1 M and 1.4 M) were prepared where the concentration of citrate was 1.2 mM. After identifying an optimum value of ammonium hydroxide (0.8 M), solutions with different concentrations of citrate (0.6 mM, 0.9 mM, 1.2 mM, 1.5 mM and 1.8 mM) were prepared. Using the optimum values obtained for these additives, a set of time growth studies was performed to monitor their effect on NR structure and length.

### 3.2. Structural Properties

**Figure 2:**
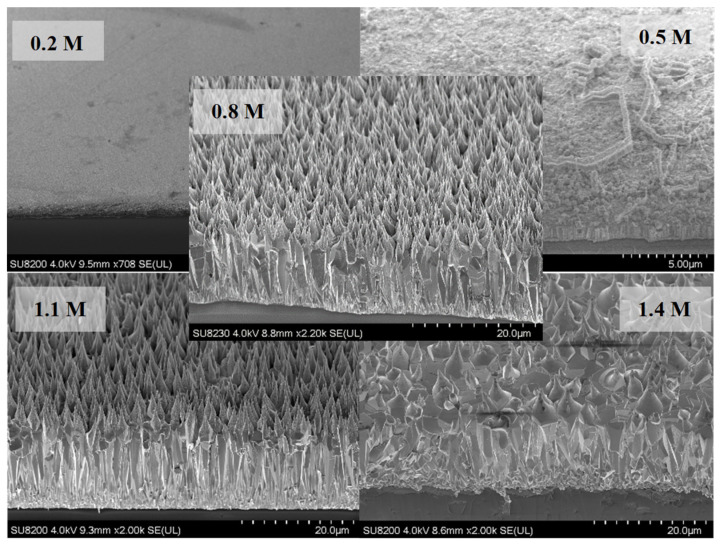
SEM views of the ZnO NR samples with different ammonium hydroxide concentrations.

Shown in Figure 2 are tilted areal and cross-sectional views of the samples along with different concentrations of ammonium hydroxide in the solution (growth time: 25 h). No growth of ZnO NRs was observed for the lowest concentration (0.2 M) while small hexagonal NRs started to grow on the surface for 0.5 M. For higher concentrations (≥0.8 M), vertical NRs that tapered towards the end were observed (Figure 2). Vertically well-aligned NRs were observed for 0.8 M and 1.1 M of which the former one had higher areal density. For the excess concentration of ammonium hydroxide (1.4 M), it was observed that the structure had deteriorated and the NR structure was no longer maintained (Figure 2 (bottom)).

**Figure 3:**
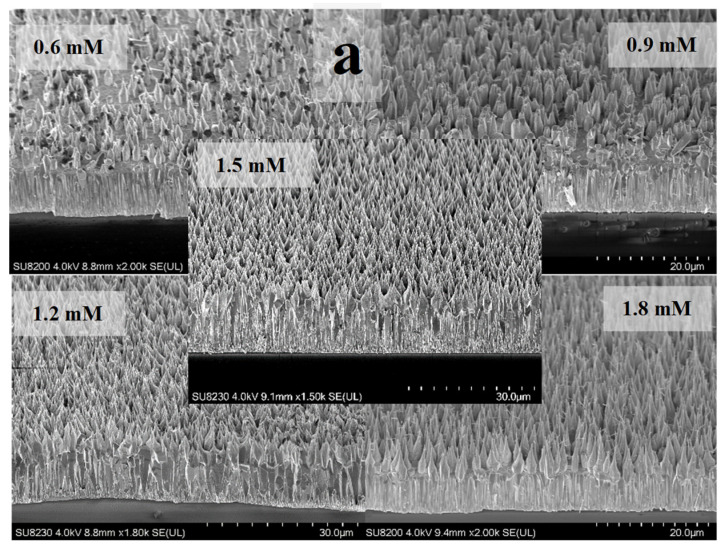

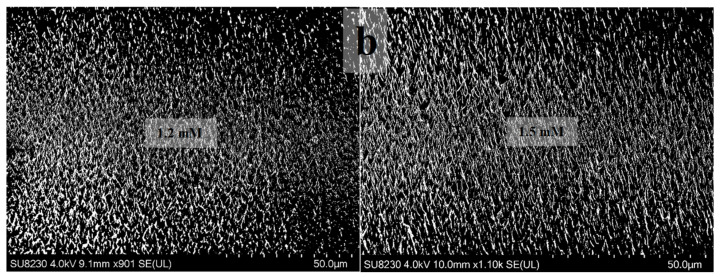
(**a**) SEM views of the ZnO NR samples with different citrate concentrations. (**b**) Black and white view of the samples adjusted to visualize the density.

**Figure 4:**
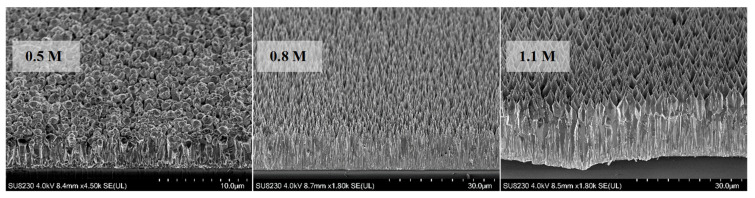
SEM views of the ZnO NRs for varying ammonium hydroxide concentrations (citrate: 1.5 mM).

Figure 3a shows the SEM views of samples containing different citrate concentrations. Samples with concentrations of 1.2 mM (~17 μm) and 1.5 mM (~20 μm) showed excellent length and surface area uniformity (the latter one being higher in density (Figure 3b)) when compared to others (Figure 3a). For the fixed concentration of 1.5 mM, a set of ZnO samples with varying ammonium hydroxide concentrations were grown. Corresponding SEM results confirmed the high density and length for the sample with 1.5 mM citrate concentration (Figure 4).

**Figure 5:**
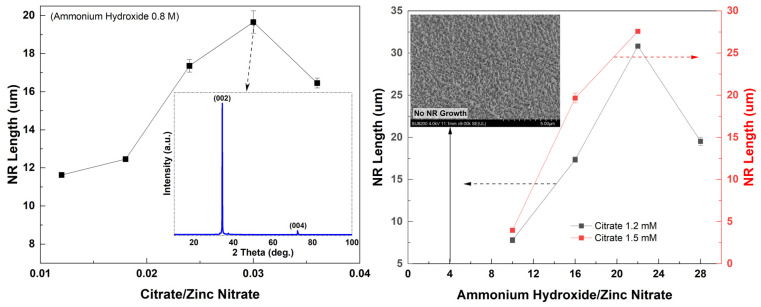
(**Left**) Variation in NR length with the citrate concentration; inset is the XRD plot for the sample with the 1.5 mM citrate. (**Right**) NR length vs. ammonium hydroxide concentration for samples with 1.2 mM and 1.5 mM citrate. (Inset) Note that ZnO NRs were not observed for the lowest concentration.

ZnO NR length gradually increased up to ~20 μm when the citrate concentration increased, except for the excess value of citrate (1.5 mM) in the solution (Figure 5 (left)). A maximum length was seen for the sample with 1.5 mM citrate, which was ~20 μm. The shown inset is the XRD pattern of the 1.5 mM citrate sample (Figure 5 (left)). The sample showed a strong (002) diffraction peak confirming that ZnO NRs were very well-aligned vertically along the c-axis. All XRD data were in excellent agreement with the standard diffraction pattern of ZnO indicating that the densely and well-aligned ZnO NRs showed a high crystalline quality and had a hexagonal wurtzite structure [47]. In general, NR length increased as the ammonium hydroxide concentration increased both for the samples with citrate concentrations of 1.2 mM and 1.5 mM for which the ZnO NRs were long and densely grown (Figure 5 (right)). While the longest NRs (>25 μm) were obtained for the 1.1 M ammonium-containing sample, the NRs were not as highly crystalline and densely grown as for 0.8 M ammonium hydroxide (Figure 2 and Figure 4). As shown in the inset for the lowest ammonium hydroxide concentration, there was no NR grown on the surface (Figure 5 (right)). Also, the length of the NRs started decreasing for the highest ammonium hydroxide concentration. This might be because the high amount of ammonium concentration leads to a higher dissolution rate of $\mathrm{Zn}{\left({\mathrm{OH}}_{4}\right)}^{2-}$ than the growth rate [30].

**Figure 6:**
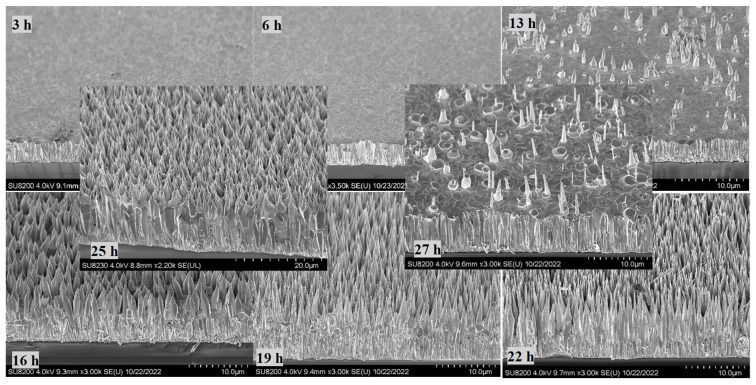
SEM views of the ZnO NRs for a time growth (1.2 mM citrate, 0.8 M ammonium hydroxide).

**Figure 7:**
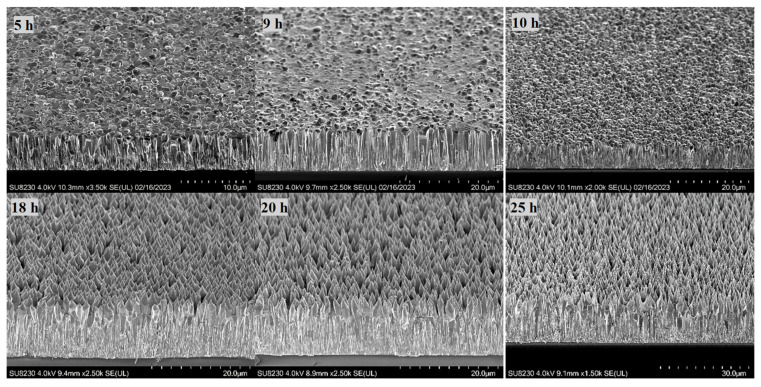
SEM views of the ZnO NRs for a time growth (1.5 mM citrate, 0.8 M ammonium hydroxide). The 5 h growth is shown here only since a similar structure was obtained for 3 h and 5 h growth periods.

**Figure 8:**
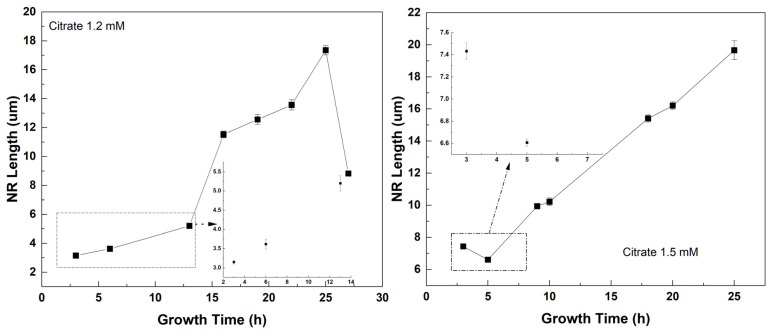
ZnO NR length vs. growth time.

To monitor the length and structure of the ZnO NRs, a set of time growth measurements was performed (Figure 6, Figure 7 and Figure 8). Concentrations of 1.2 mM and 1.5 mM citrate and 0.8 M ammonium hydroxide were selected as they constituted the higher quality crystalline structure and well-aligned dense NRs. First, ZnO NRs were synthesized for a time period ranging 3–27 h using 1.2 mM citrate and 0.8 M ammonium hydroxide. SEM views of the samples are shown in Figure 6. It was seen that the ZnO NRs maintained a hexagonal structure on top of the surface until 13 h, then it started tapering (Figure 6). For the growth time greater than 25 h, i.e., 27 h, the solution contained very low nutrient concentrations, thus, the length of the ZnO NRs started decreasing and nonuniform NRs started to grow (Figure 8 (left)). Then, the ZnO NRs were synthesized for a period ranging 3–25 h using 1.5 mM citrate and 0.8 M ammonium hydroxide concentrations (Figure 7). A similar observation with a slight difference in the tapering process was observed for this set of measurements. ZnO NRs started tapering from 9 h and by the growth time of 10 h the surface seemed tapered (Figure 7). Since the nutrients deplete in the solution, ZnO NRs might start tapering towards the end of the growth. For shorter growth times, the nutrients depletion rate was much lower than that for longer growth times. Therefore, tapering was less below 10 h time growth and was much more after a certain time of growth (Figure 6 and Figure 7).

**Figure 9:**
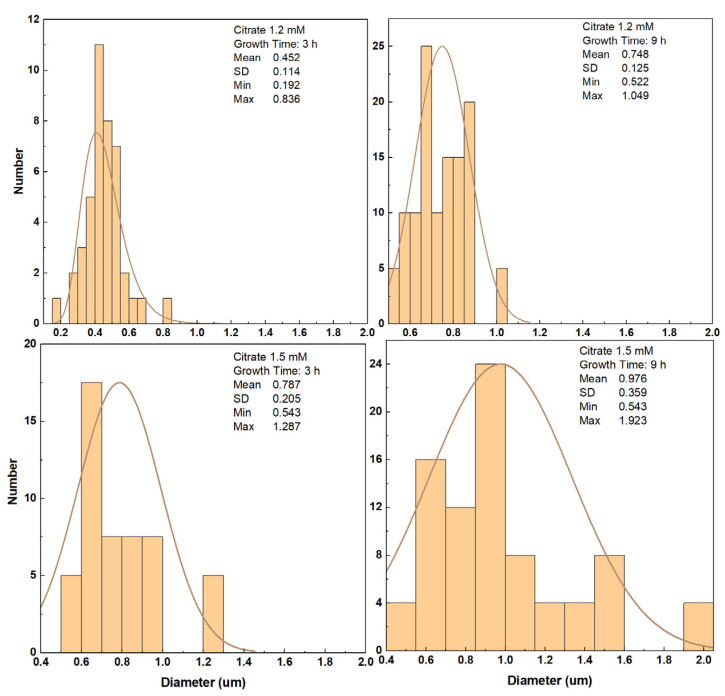
Dependence of ZnO NR diameters for different citrate concentrations. Nontapered ZnO NRs were chosen to measure the diameter.

The ammonium hydroxide is shown to increase the growth rate in the solution. However, the presence of ions in the solution are believed to adsorb on the ZnO basal planes such that axial growth along the c-axis is inhibited while the lateral growth is promoted in addition to the axial growth [48,49]. Thus, relatively wider NRs are expected with the increase in the citrate concentration (Figure 9).

### 3.3. Optical Properties

**Figure 10:**
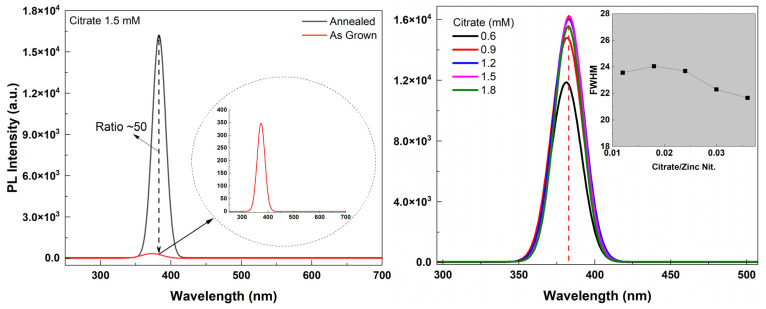
PL spectra of an annealed vs. an as-grown sample (**left**). PL spectra (fitted) for samples with different citrate concentrations (**right**).

Annealing the samples in a 10% H_2_ atmosphere enhanced the crystalline quality of the NRs and resulted in a very strong UV emission as the peak intensity ratio of annealed/as-grown samples was ~50 (Figure 10 (left)). The PL spectra (Gauss fitted) of annealed (in forming gas) samples with different citrate concentrations are shown in Figure 10 (right). Also shown in the inset in Figure 10 (right) is the FWHM vs. citrate concentration. It was observed that the NBE UV peak intensity increased until the longest length of the NRs was reached (citrate: 1.5 mM). Also, a red shift in the peak position was observed as the citrate concentration increased (as shown in Figure 10 (right) by the dashed red line). This might be due to the high carrier concentration which makes the Burstein–Moss (BM) effect dominant in the PL spectra. It was reported that the excitation density increases as the diameter decreases and, with a high carrier concentration, it can cause the enlargement of the bandgap; thus, a blue shift in UV luminescence with the decreasing diameter of the NR could be observed [50]. Our results support this finding as the diameter of the NRs increased with increasing citrate concentration. Therefore, a red shift instead of blue shift in UV PL emission was observed with the increase in diameter of the NRs.

**Figure 11:**
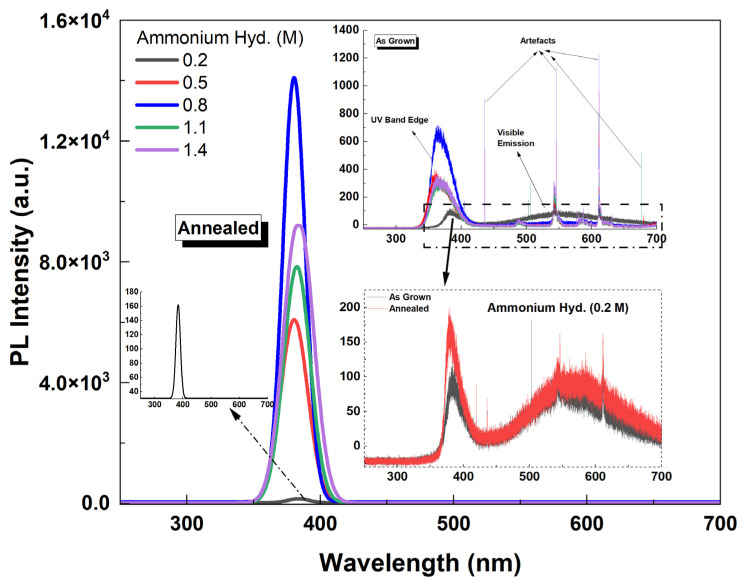
PL spectra (fitted) of ZnO NR samples with different ammonium hydroxide concentrations. Right inset shows the raw PL spectra (not fitted) of as-grown ZnO NR samples along with the as-grown vs. annealed sample for the lowest ammonium hydroxide concentration.

Shown in Figure 11 is the PL spectra (fitted) of ZnO NR samples grown with different ammonium hydroxide concentrations. It was found that the intensity of the UV emission intensity increased as the concentration increased up to 0.8 M (as the NR length also increased). However, for further increasing NR length (ammonium hydroxide: 1.1 M), the emission intensity began to decrease. This might be due to the lower crystalline quality and less dense array of NRs for 1.1 M than the 0.8 M (Figure 2). Also, shown as an inset in Figure 11 (right) is the PL spectra (not fitted) for as-grown ZnO NR samples. It was observed that without annealing, the UV PL emission was significantly weaker and a visible emission band centered in the green region (~550 nm) was observed for the sample with 0.2 M ammonium hydroxide. As we used citrate in our growth solution, the visible emission was significantly suppressed. As mentioned before, it promotes lateral growth rather than axial growth, therefore, relatively wider NRs; thus, large diameters were obtained. It was reported before that the NBE UV emission increases when the diameter of the nanowires increases [51], however, it should be noted that the sample with 0.2 M ammonium hydroxide did not result in any NR growth (Figure 2 and Figure 5). Therefore, the hexagonal structure was not maintained and the crystal quality was significantly lower. This might be the reason that the defect-related visible emission occurred for this sample. In addition, it was seen that while annealing slightly enhanced the UV emission, the visible emission band remained comparably high in the structure for the sample with 0.2 M ammonium hydroxide (Figure 11 (bottom right inset)).

**Figure 12:**
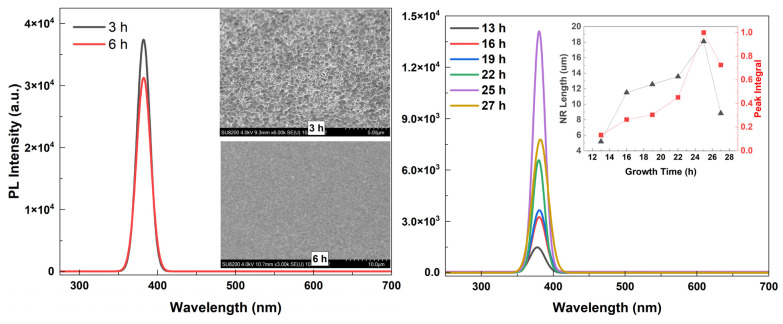
Typical PL spectra of samples for different growth times (citrate: 1.2 mM). (**Left**) ZnO NRs with nontapered structure (inset shows the SEM image of nontapered NRs). (**Right**) ZnO NRs with tapered structure (inset represents the variation in NR length and normalized PL peak integral with the growth time).

Figure 12 shows the typical PL spectra of samples grown for different growth times (citrate: 1.2 mM). The PL spectra are divided into two regions: the first region refers to ZnO NRs with no tapering (left) and the second region to ZnO NRs exhibiting tapering towards the end of the structure (right). ZnO NRs with no tapering resulted in a higher UV emission relative to the tapered ZnO NRs. This was attributed to a very dense structure as implied by the mirror-like reflection property (Figure 12 (left)). However, as is discussed later in the manuscript, these nontapered NRs have a very short length (<4 μm), which results in a significantly low scintillation yield. When it comes to the second region, the UV PL intensity was significantly enhanced for the longer growth times as long as the crystalline structure was maintained (Figure 12 (right)). Results clearly confirm that as the growth time increases the length of the ZnO NRs gradually increases, and consequently the crystallization quality improves [47].

### 3.4. Scintillation Properties

**Figure 13:**
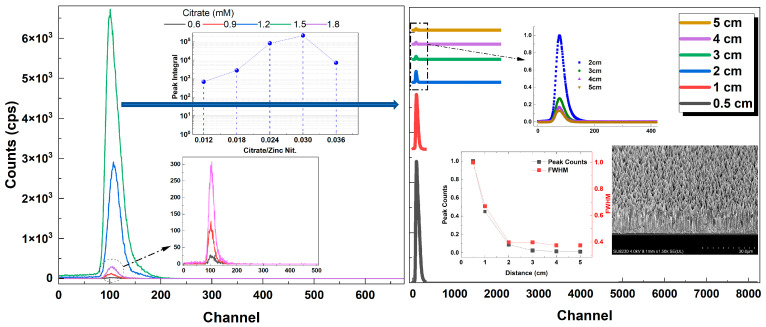
PHD of the alpha response of ZnO NR samples with different citrate concentrations (**left**). Shown in the top inset is the peak area integral vs. citrate concentration (**left**). Alpha response vs. source–detector distance of the ZnO NR sample with highest alpha response (**right**). Shown insets are the peak counts and FWHM vs. distance and the tilted cross-sectional SEM image (**right**). All counts are background corrected net counts.

As mentioned earlier in the manuscript, ZnO is a good candidate as a scintillator with its great properties, such as high stopping power, fast decay time, radiation tolerance and high NBE UV emission. The ZnO NR samples grown in this study were tested in terms of alpha particle response using an Am-241 alpha test source. The pulse height distribution (PHD) spectra of the ZnO NR samples with different citrate concentrations are shown in Figure 13 (left). Samples with citrate concentrations of 1.2 mM and 1.5 mM showed relatively higher alpha responses, while the latter one had a significantly higher response amongst all the samples investigated. Further measurements with respect to the source–detector distance, keeping the counting time relatively longer, were performed on the ZnO NR sample exhibiting the highest alpha response (Figure 13 (right)). A good alpha response with respect to the source–detector distance was obtained. The maximum distance was set to 5 cm as the alpha particles were easily absorbed in the air. As expected, the peak counts and the FWHM gradually decreased and then became almost constant as the distance increased (Figure 13 (right, inset)).

**Figure 14:**
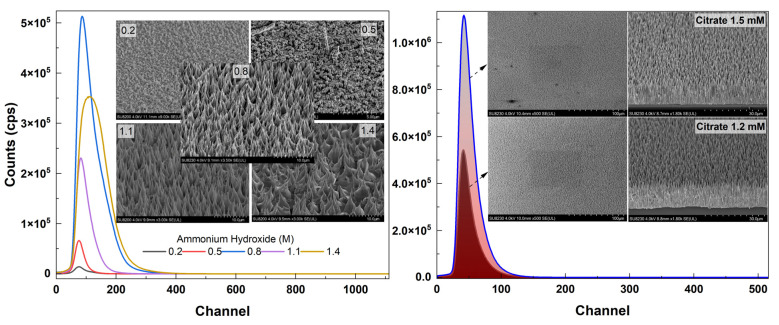
Alpha responses of ZnO NR samples with different ammonium hydroxide concentrations (**left**). Shown inset is the surface view of the samples (**left**). Alpha responses of the samples with citrate concentrations of 1.2 mM and 1.5 mM (**right**). Insets are the low-magnification top view and high-magnification side view images (**right**).

Figure 14 (left) shows the alpha response (fitted) of samples with varying ammonium hydroxide concentrations. It was seen that for relatively low ammonium hydroxide concentrations, the alpha response was weak. This is because they have either no NRs grown on the surface or have a very short length (<5 μm) (Figure 14 (left, inset)). If the ZnO NRs are not thick enough to fully stop the energetic alpha particles (~5.5 MeV), the subsequent scintillation in the material will be much less since the energy transfer of particles will be small. However, the crystalline quality and the UV PL emission are also important in getting good scintillation for these types of materials. For the ZnO NR samples with the highest alpha response (ammonium hydroxide: 0.8 M), a set of high-surface-area samples with citrate concentrations of 1.2 mM and 1.5 mM, whose alpha responses were relatively high compared to other samples, were grown and annealed. The alpha response results of these samples are shown in Figure 14 (right). It was seen that both samples had very homogenously distributed NR surfaces and very well-aligned NRs with large thickness resulting in significantly high alpha responses (Figure 14 (right)). The ZnO NR sample with 1.5 mM citrate was found to have the highest alpha response amongst all other samples.

**Figure 15:**
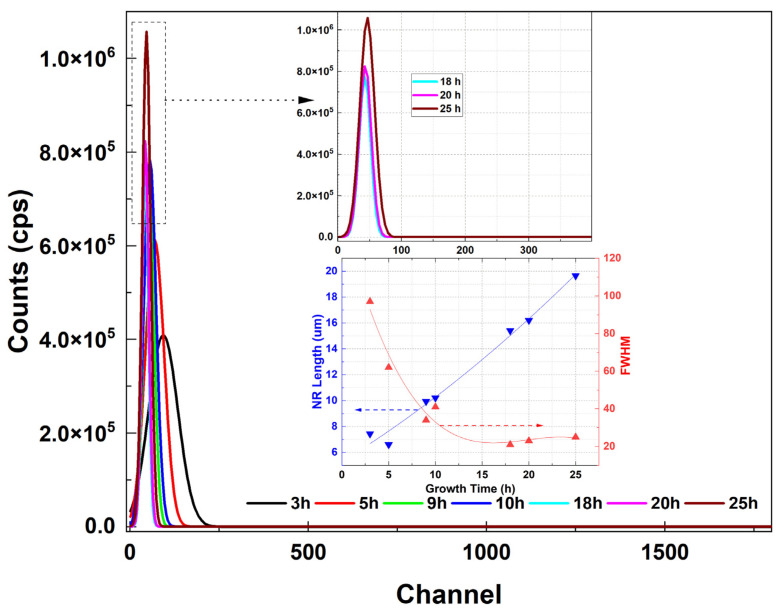
Alpha response (fitted) of the ZnO NRs vs. growth time. Bottom inset shows NR length and FWHM vs. growth time.

For the ZnO NRs having the highest alpha and PL responses (citrate: 1.5 mM), a set of alpha response measurements vs. growth time were carried out (Figure 15). It was seen that the alpha responses gradually increased for the longer growth times (Figure 15). This is because the crystalline quality improves as the growth time increases and the energy deposition (scintillation) of the interacting alpha particles is much higher for the larger thickness of ZnO NRs. The inset figure shows the NR length and FWHM vs. growth time confirming a decrease in FWHM with the increasing growth time.

**Figure 16:**
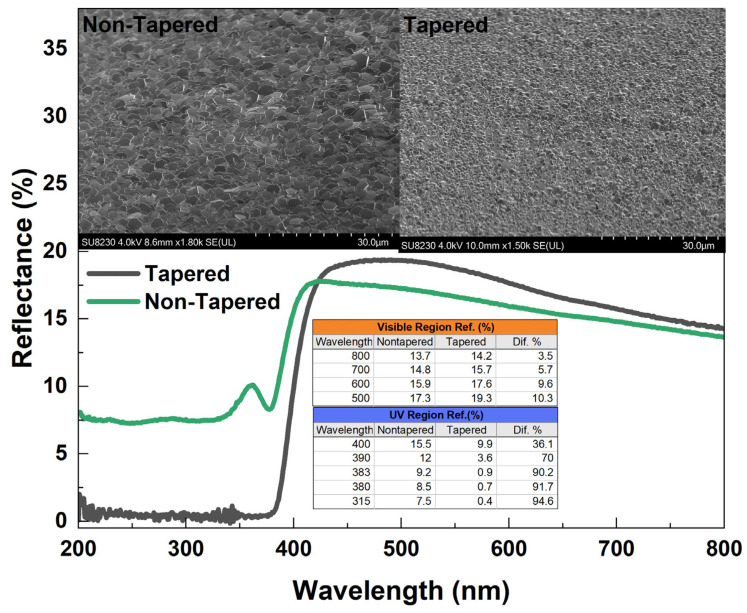
Reflectance spectra of ZnO NRs.

Figure 16 represents the reflectance spectra of ZnO NRs with and without tapering. The growth time was arranged (~10 h) such that tapered and nontapered ZnO NRS had almost the same length. The nontapered NRs were obtained in a two-cycle growth while the tapered NRs were obtained in one cycle of growth. Both ZnO NRs exhibited the same relatively high reflectance, but less than 20% in the visible–near-IR range. However, as shown in the inset table, the reflection was much less for both of the ZnO NR samples in the UV region. In fact, the tapered ZnO NRs showed an excellent antireflection feature when compared to the nontapered ZnO NRs in the UV region (Figure 16 (inset table)). A wavelength dependence was strongly observed for tapered NRs and a fluctuation in spectra was observed for nontapered ZnO NRs at approximately 360 nm (Figure 16). This fluctuation may refer to interference fringes and can be eliminated in a tapered surface resulting in a rough ZnO–air interface [5]. It can be seen that the tapered ZnO NRs can help the light absorption into the structure and, thus, enhance the scintillation efficiency and increase light coupling into the output device.

## 4. Conclusions

In the present work, long and well-aligned hexagonal ZnO NRs were grown using citrate and ammonium hydroxide as additives in one growth cycle. The effects of the additives involved in the growth procedure, i.e., ammonium hydroxide and sodium citrate, were studied in terms of the morphological, optical and scintillation properties of the ZnO nanostructures. Dependence of PL and scintillation on ZnO NR length and additive concentration were also discussed. ZnO NRs grown on a silica substrate, whose UV transmission was found to be better than glass, showed high-quality structural and optical properties. SEM images revealed a better crystalline structure and a high alignment if the growth time was increased to 25 h. The addition of sodium citrate along with ammonium hydroxide significantly reduced defects and strongly enhanced the intrinsic NBE UV emission intensity at ~380 nm. Also, a red shift in the NBE UV emission was observed as the citrate concentration increased. In order to obtain high-quality nanostructures, samples were annealed in a forming gas. Anneals in a forming gas atmosphere enhanced the emission of the UV peak by reducing defects in the nanostructure. Scintillation measurements revealed that the homogenously distributed and very well-aligned tapered ZnO NRs with relatively large thickness resulted in significantly higher alpha responses. ZnO NRs tapered towards the end were found to be highly antireflective, especially in the UV region. Therefore, tapered well-aligned ZnO NRs could act as a good light-trapper and absorber, thus, enhancing the scintillation efficiency by increasing scintillation light coupling in the sample to detector interface.

## Data Availability

All data are contained within the article.
